# Policy statement of enteral nutrition for preterm and very low birthweight infants

**DOI:** 10.1111/ped.14067

**Published:** 2020-02-05

**Authors:** Katsumi Mizuno, Toshiaki Shimizu, Shinobu Ida, Setsuko Ito, Mikako Inokuchi, Toshihiro Ohura, Akihisa Okumura, Masanobu Kawai, Toru Kikuchi, Motoichiro Sakurai, Shigetaka Sugihara, Mitsuyoshi Suzuki, Kimitaka Takitani, Daisuke Tanaka, Sotaro Mushiake, Nobuo Yoshiike, Hiroko Kodama, Kazuo Okada, Chiharu Tsutsumi, Mitsuhiko Hara, Yoshio Hanawa, Kazue Kawakami, Hiroaki Inomata, Tatsuya Oguni, Yuko Bito, Keiichi Uchida, Akihide Sugiyama

**Affiliations:** ^1^ Director Committee on Nutrition The Child Health Consortium of Japan Tokyo Japan; ^2^ Chair Committee on Nutrition The Child Health Consortium of Japan Tokyo Japan; ^3^ Board Committee on Nutrition The Child Health Consortium of Japan Tokyo Japan

**Keywords:** donor human milk, exclusive human milk-based diet, human milk, human milk bank

## Abstract

1. For preterm and very low birthweight infants, the mother’s own milk is the best nutrition. Based on the latest information for mothers who give birth to preterm and very low birthweight infants, medical staff should encourage and assist mothers to pump or express and provide their own milk whenever possible.
2. If the supply of maternal milk is insufficient even though they receive adequate support, or the mother’s own milk cannot be given to her infant for any reason, donor human milk should be used.
3. Donors who donate their breast milk need to meet the Guideline of the Japan Human Milk Bank Association.
4. Donor human milk should be provided according to the medical needs of preterm and very low birthweight infants, regardless of their family’s financial status.
5. In the future, it will be necessary to create a system to supply an exclusive human milk‐based diet (EHMD), consisting of human milk with the addition of a human milk‐derived human milk fortifier, to preterm and very low birthweight infants.

## Introduction

For preterm infants, especially for very low birthweight infants and high‐risk neonates with gastrointestinal and heart disease, the first choice for enteral feeding is their mother’s own milk. To be able to express the mother’s own milk as early as possible, obstetrics and neonatal intensive care unit (NICU) staff should provide significant support for mothers based on scientific evidence.

Some mothers may not be able to provide enough breast milk even with sufficient support or feed it for various reasons. The European Society for Paediatric Gastroenterology, Hepatology and Nutrition (ESPGHAN) and the American Academy of Pediatrics (AAP) recommend that donor human milk^1^ that is pasteurized and managed by established human milk banks is provided to the baby^1^, ^2^ in such cases. In the neonatal intensive care units (NICUs) in Japan, when the mother’s own milk cannot be obtained, “donated unpasteurized breast milk”^2^ that is not pasteurized is sometimes used. (Donor human milk is breast milk that has been processed in an established human milk bank and proved to be sterile by culture tests. Donated unpasteurized breast milk is breast milk provided by women other than the infant’s mother that is frozen but not pasteurized. Bacterial tests are not usually conducted, and the women's condition when expressing – e.g. with or without taking cold medicine, smoking, and drinking – is also unknown.)

## Policy statement

However, we have to consider the risks of “donated unpasteurized breast milk” because outbreaks of multidrug‐resistant bacteria via breast milk have been observed.^3^ In addition, while the Pediatric Society has issued a statement regarding Advocacy on Human Breast Milk Traded over the Internet, even healthy infants should avoid receiving breast milk from women whose health status is unknown.^4^

Several reviews have been published indicating that donor human milk provided from established human milk banks is useful for preventing complications such as neonatal necrotizing enterocolitis^1^, ^5^, ^6^, ^7^, ^8^, ^9^, ^10^, ^11^, ^12^ in preterm infants. As a result, human milk banks have been established in countries around the world, including Asia, and systems that allow any NICU to use donor human milk if needed have been created. The Japan Human Milk Bank Association (JHMBA) was established in 2017, and the association is in the process of creating a system to provide donor human milk when infants cannot have their mothers’ own milk. If mothers cannot provide enough breast milk or feed it even with appropriate support, using donor human milk provided by human milk banks is also recommended in Japan. Some mothers may be discouraged because they have to use donor human milk for their infants and they feel that lack of lactation is their own fault. Medical staff should pay attention to these psychological considerations for the mother and tell her that it is a "treatment" in a broad sense and that it is possible to feed their own milk after expressing successfully. While using donor human milk, support and consideration of the mothers' feelings and emotions by cooperation with staff of various occupations are needed.

In preterm and very low birthweight infants, as feeding breast milk alone leads to a to a lack of the nutrients needed for the growth and development of the infants, human milk fortifier is usually added. However, bovine milk‐derived fortifier increases enteral feeding intolerance,^13^ onset of milk allergy,^14^ fatty acid calcium stone formation,^15^ etc. An exclusive human milk‐based diet (EHMD) is human milk with the addition of a human milk‐derived human milk fortifier, and substances derived from bovine milk are not added at all. In recent years, there have been many reports on the benefits of EHMDs, and it will be necessary for Japan to create systems supplying EHMDs.

1. The mother’s own milk is the best nutrition for preterm and very low birthweight infants, and NICU staff should recommend and support breastfeeding.
2. The next option if the mother’s own breast milk is not obtainable or sufficient is donor human milk that has been pasteurized in established human milk banks.
3. In the future, it will be desirable to provide EHMD, human milk supplemented with human milk‐derived human milk fortifier, to preterm and very low birthweight infants.

## Background and content of this policy statement

The best nutrition not only for preterm and very low birthweight infants but also for newborns and infants in general is their mothers’ own milk. Starting breastfeeding of preterm infants with the mother’s milk after birth has various advantages, such as a preventive effect on neonatal necrotizing enterocolitis, chronic lung disease, and acquired sepsis, as well as improvement of neurodevelopmental outcomes thereafter. It has been reported that even preterm mothers are able to enhance lactation by starting to express within several hours after delivery and expressing by using a combination of a hospital‐grade electric pump and their hands. Therefore, based on current scientific knowledge, it is desirable that all NICUs provide breastfeeding support.

There are some NICUs that do not feed infants anything until their mother’s own milk is obtained. In that case, the problem is how long the infants have to wait before the breast milk arises as fasting may cause atrophy of the gastrointestinal mucosa and bacterial translocation in the infants. In recent years, it has been reported that there has been an improvement in short‐term prognosis in very low birthweight infants by starting enteral nutrition as soon as possible after birth. Therefore, there is a tendency to start enteral nutrition within 24 h of birth^13^. In order to start enteral nutrition in a situation without the mother’s own milk, we often use infant formula for low birthweight infant or “donated unpasteurized breast milk” that is non‐pasteurized milk. The formula and “donated unpasteurized breast milk” have disadvantages because the formula increases the risk of neonatal necrotizing enterocolitis and the donated unpasteurized breast milk has the risk of viral and bacterial infection. On the other hand, donor human milk that has been appropriately managed and pasteurized in established human milk banks is considered to be generally safe because microorganisms such as viruses and bacteria are killed.^16^, ^17^, ^18^, ^19^, ^20^

From the above, in order to standardize the safe enteral nutrition of preterm and very low birthweight infants after birth, we would like to recommend the use of donor human milk provided by human milk banks.

When establishing enteral nutrition, we need to use human milk fortifier. It has been reported that using a human milk‐derived human milk fortifier not derived from bovine milk provides various advantages, such as earlier establishment of enteral nutrition, a shortened period of parenteral nutrition, and a reduction in the complications often seen in very premature infants (e.g. neonatal necrotizing enterocolitis, chronic lung disease, and retinopathy of prematurity), and a shortened period of hospitalization.^20^, ^21^ In future, we will also have to create systems for supplying EHMD in Japan.

## Human milk bank

Current status of human milk banks worldwide and history of establishment of the bank in Japan. There is a movement worldwide to establish human milk banks, and more than 600 banks operate and manage donor human milk in over 50 countries. Twenty‐seven non‐profit human milk banks that are members of the Human Milk Banking Association of North America have already been established, and three additional banks were newly established over the past year. Human milk banks have also established in Asia (e.g. China, Korea, Taiwan, Singapore, Vietnam, India, and the Philippines). In Japan, the Guideline for the Establishment of a Breast Milk Bank was created as a shared research project of the Health and Labor Science Research (Next‐generation Development Basic Research Project for Overcoming Children's Diseases: Study of Prevention of Mother‐to‐Child HTLV‐1 Transmission: Cohort Study among Offspring of HTLV‐1 Infected Mothers) over three years from April 2014 to March 2017. After approval by the Ethics Committee of the Showa University Koto Toyosu Hospital in 2014, a draft of the Standard Operating Procedure was created based on the guidelines of the Human Milk Banking Association of North America – that is, the Guidelines for the Establishment and Operation of a Donor Human Milk Bank 2013. It was also created by consulting the guidelines of the European Milk Bank Association (i.e. Guidelines for the Establishment and operation of a donor human milk bank) and the National Institute for Health and Clinical Excellence (i.e. Guidelines for the Establishment and operation of human milk banks in the UK).^22^, ^23^, ^24^ Based on this draft, comments from experts in the fields of food hygiene, hazard analysis and critical control points (HACCP), nutrition, and microbiology, and neonatal doctors (Director, the Japan Society of Perinatal and Neonatal Medicine) were gathered, and then the first Standard Operating Procedure was created. After revisions following discussions with research members of the Ministry of Health, Labor and Welfare (MHLW), the current Standard Operating Procedure was prepared. Then, the operation of a breast milk bank was started at Showa University Koto Toyosu Hospital, in accordance with the Standard Operating Procedure.

With that background, the Japan Human Milk Bank Association was established in May 2017. Here are descriptions of the operation of the bank:

### Registration of donors

Performs medical checkups and serum screening tests (HIV, HTLV‐1. HBV, HCV, syphilis), etc., and explains how to express, preserve, and deliver breast milk. In general, as the health condition of women who donate “donated unpasteurized breast milk” is unchecked, there is variability in the quality of the shared milk.

### Sterilization of breast milk

There are several methods to pasteurize breast milk. As holder pasteurization at 62.5 °C for 30 min is the standard method in Europe and the USA, the Japan Human Milk Bank Association also adopted the same pasteurization conditions.

### Providing donor human milk

The Japan Human Milk Bank Association provides donor human milk only to neonatal intensive care facilities. The donor human milk has a label with a batch number and expiration date. This batch number is linked to the donor.

### Safety of donor human milk

Human milk is a biological product; therefore, whether from an infant’s own mother or a donor mother, there will always be concerns about contamination. Possible contaminants are infectious agents such as viruses and bacteria, and drugs they have taken. There is a risk of infection from "donated unpasteurized breast milk", even if it is the breast milk from a woman who is negative for HIV, HTLV‐1, HBV, HCV, and syphilis in screening tests for donors. Holder pasteurization has been shown to be effective in removing infectious contaminants, and there has been no report that infections such as hepatitis or HIV have been caused by donor milk provided by a previously established breast‐milk bank.^2^

### Growth with human milk‐derived human milk fortifier

When donor human milk is used, human milk fortifiers should be added to lead to appropriate growth of preterm and very low birthweight infants.

### Cost burden

Whether the cost of donor milk is borne by the hospital or the infant’s family is a major issue. Society‐wide action is required to make it possible for all babies who need donor human milk to receive it. Feeding with donor human milk can lead high‐risk children to better health.^25^ Whether or not preterm and very low birthweight infants use donor human milk should be decided upon based on medical judgment, not their family's financial status.

## Future issues

Organization of human milk banks: According to the results of a questionnaire survey obtained from 168 NICUs in Japan in 2016, most facilities prioritized provision of mother’s own milk to very low birthweight infants whose birthweight is less than 1,000 g as a method to supply calories and nutrients for 1 month after birth. When insufficient breast milk was obtained, they provided an infant formula for low birthweight infants.^26^ The usability of donor human milk has been reported in countries where human milk banks have been established, and new human milk banks are being established every year to make donor human milk accessible. It was revealed that 75% of NICUs in Japan considered human milk banks to be necessary.^27^ As mentioned above, the Japan Human Milk Bank Association was established in 2017, and NICUs using donor human milk have increased since 2018. We have to inform pediatric and neonatal care personnel about the utility of donor human milk provided by the human milk bank and establish as many human milk banks as necessary to meet domestic demand.

Standardized enteral nutrition for preterm and very low birthweight infants: As a result of meta‐analysis, it has been shown that neonatal necrotizing enterocolitis is reduced by about 1/5 when standardized enteral nutrition of preterm and very low birthweight infants is used.^28^ Using standardized enteral nutrition with donor human milk instead of low‐birthweight infant formula should be recommended when mothers cannot express breast milk or feed it.

Supplying EHMD: Prolacta Bioscience, Inc. In the United States, there is one company that manufactures human milk‐derived human milk fortifier by using a process such as ultrafiltration from donor human milk. There are four types of fortifiers (i.e. +4, +6, +8, and + 10H^2^MF), and additional proteins and calories can be provided according to each infant’s condition. It allows all infants to receive optimal milk. On the other hand, it is unrealistic to purchase this at the expense of the patient's family because of the high cost of the fortifier. However, as described above, EHMD has been shown to reduce the complications of preterm and very low birthweight infants.^20^ It might reduce the effort required for respiratory circulation management, parenteral nutrition management, and fundus examination. As a result, providing EHMD to infants makes it possible to reduce medical expenses. A publication on health economics states that there is no overall loss to facilities, even if the facilities pay for the human milk‐derived human milk fortifier.^29^ The Canadian Premature Babies Foundation requires the Canadian government to provide EHMD for all premature infants in Canada (https://www.communitywire.ca/en/2016-09-28/breast-milk-every-preemie).

Japan's neonatal care is of the highest quality in the world. Considering the problem of the declining birthrate in Japan, it is important for pediatricians to continue their efforts to improve the long‐term prognosis for each premature infant. We must appeal to the Japanese society and the government so that EHMD can be supplied regardless of the patient's family's financial status.

## Disclosure

The authors declare no conflicts of interest.

## References

[1] Arslanoglu S , Corpeleijn W , Moro G *et al* Donor human milk for preterm infants : current evidence and research directions. J. Pediatr. Gastroenterol. Nutr. 2013; 57 : 535–42. 24084373 10.1097/MPG.0b013e3182a3af0a

[2] Committee on Nutrition, American Academy of Pediatrics Policy Statement . Donor Human Milk for the high‐risk infants : preparation, safety, and usage options in the United States. 2017 Pediatrics; 139: e20163440. 27994111 10.1542/peds.2016-3440

[3] Nakamura K , Kaneko M , Abe Y *et al* Outbreak of extended‐spectrum β‐lactamase‐producing Escherichia coli transmitted through breast milk sharing in a neonatal intensive care unit. J. Hosp. Infect. 2016; 92: 42–6. 26238662 10.1016/j.jhin.2015.05.002

[4] Advocacy on Human Breast Milk Traded over the Internet , [Cited 2019 April 15]. Available from:https://www.jpeds.or.jp/modules/news/index.php?content_id=167.

[5] Landers S , Hartmann BT . Donor human milk banking and the emergence of milk sharing. Pediatr. Clin. North Am. 2013; 60: 247–60. 23178068 10.1016/j.pcl.2012.09.009

[6] Arslanoglu S , Ziegler EE , Moro GE . World Association of Perinatal Medicine Working Group on Nutrition. Donor human milk in preterm infant feeding: evidence and recommendations. J. Perinat. Med. 2010; 38: 347–51. 20443660 10.1515/jpm.2010.064

[7] Bertino E , Giuliani F , Baricco M *et al* Benefits of donor milk in the feeding of preterm infants. Early Hum. Dev. 2013; 89 (suppl 2): S3–6. 10.1016/j.earlhumdev.2013.07.008 23932110

[8] Quigley M , McGuire W . Formula versus donor breast milk for feeding preterm or low birth weight infants. Cochrane Database Syst. Rev. 2014; 4: CD002971. 10.1002/14651858.CD002971.pub3 24752468

[9] Section on Breastfeeding . Breastfeeding and the use of human milk. Pediatrics 2012; 129: e827–41. Available at : http://www.pediatrics.org/cgi/content/full/129/3/e827 22371471 10.1542/peds.2011-3552

[10] Sullivan S , Schanler RJ , Kim JH *et al* An exclusively human milk‐based diet is associated with a lower rate of necrotizing enterocolitis than a diet of human milk and bovine milk‐based products. J. Pediatr. 2010; 156: 562–67. e1. 20036378 10.1016/j.jpeds.2009.10.040

[11] Cristofalo EA , Schanler RJ , Blanco CL *et al* Randomized trial of exclusive human milk versus preterm formula diets in extremely premature infants. J. Pediatr. 2013; 163: 1592–1595. e1. 23968744 10.1016/j.jpeds.2013.07.011

[12] Kantorowska A , Wei JC , Cohen RS *et al* Impact of donor milk availability on breast milk use and necrotizing enterocolitis rates. Pediatrics 2016; 137: e20153123. 26908696 10.1542/peds.2015-3123 PMC4771129

[13] Sandhu A , Fast S , Bonnar K *et al* Human‐based human milk fortifier as rescue therapy in very low birth weight infants demonstrating intolerance to bovine‐based human milk fortifier. Breastfeed. Med. 2017; 12: 570–73. 28777664 10.1089/bfm.2017.0017 PMC5684660

[14] Oba K , Obana N , Hayashi K *et al* A very low birth weight infant diagnosed with gastrointestinal food allergy by human milk fortifier. J. Jpn. Soc. Premature Newborn Med. 2014; 26: 320–23.

[15] Murase M , Miyazawa T , Taki M *et al* Development of fatty acid calcium stone ileus after initiation of human milk fortifier. Pediatr. Int. 2013; 55: 114–16. 23409991 10.1111/j.1442-200X.2012.03630.x

[16] Human Milk Banking Association of North America . Guidelines for Establishment and Operation of a Donor Human Milk Bank, 16th edn Human Milk Banking Association of North America, Fort Worth, TX, 2011.

[17] Czank C , Prime DK , Hartmann B *et al* Retention of the immunological proteins of pasteurized human milk in relation to pasteurizer design and practice. Pediatr. Res. 2009; 66: 374–79. 19581827 10.1203/PDR.0b013e3181b4554a

[18] Landers S , Updegrove K . Bacteriological screening of donor human milk before and after Holder pasteurization. Breastfeed. Med. 2010; 5: 117–21. 20509779 10.1089/bfm.2009.0032

[19] Terpstra FG , Rechtman DJ , Lee ML *et al* Antimicrobial and antiviral effect of high‐temperature short‐time (HTST) pasteurization applied to human milk. Breastfeed. Med. 2007; 2: 27–33. 17661617 10.1089/bfm.2006.0015

[20] Hair AB , Peluso AM , Hawthorne KM *et al* Beyond necrotizing enterocolitis prevention : improving outcomes with an exclusive human milk‐based diet. Breastfeed. Med. 2016; 11: 70–4. 26789484 10.1089/bfm.2015.0134 PMC4782036

[21] HMBANA . Guidelines for the establishment and operation of a donor human milk Bank.2013. 10.3109/14767058.2010.512414 20840052

[22] EMBA . Guidelines for the establishment and operation of a donor human milk bank. J. Maternal‐Fetal Neonatal Med. 2010; 23 (S2): 1–20. 10.3109/14767058.2010.512414 20840052

[23] de Segura AG , Escuder D , Montilla A *et al* Heating‐induced bacteriological and biochemical modifications in human donor milk after holder pasteurisation. J. Pediatr. Gastroenterol. Nutr. 2012; 54: 197–203. 21921811 10.1097/MPG.0b013e318235d50d

[24] The National Institute for Health and Clinical Excellence . [Cited 2019 April 15]. Available from:http://publications.nice.org.uk/donor-milk-banks-the-operationof-donor-milk-bank-services-cg93/guidance.

[25] Perrine CG , Schanlon KS . Prevalence of use of human milk in US advanced care neonatal units. Pediatrics 2013; 131: 1066–71. 23669517 10.1542/peds.2012-3823 PMC4535053

[26] Suzuki M , Itabashi K .Questionnaire results and commentaries: Amino acid and blood glucose. The 16th Newborn nutrition forum, abstracts. 2016 : 4–10.

[27] Mizuno K , Sakurai M , Itabashi K . Necessity of human milk banking in Japan: Questionnaire survey of neonatologists. Pediatr. Int. 2015; 57: 639–44. 25728542 10.1111/ped.12606

[28] Jasani B , Patole S . Standarized feeding regimen for reducing necrotizing enterocolitis in preterm infants: An updated systematic review. J. Perinatol. 2017; 37: 827–33. 28358382 10.1038/jp.2017.37

[29] Assad M , Elliott MJ , Abraham JH . Decreased cost and improved feeding tolerance in VLBW infants fed an exclusive human milk diet. J. Perinatol. 2016; 36: 216–20. 26562370 10.1038/jp.2015.168

